# Unraveling metabolic shifts in peach under agrochemical treatments during flower bud endodormancy in the context of global warming

**DOI:** 10.3389/fpls.2025.1502436

**Published:** 2025-03-19

**Authors:** Jesús Guillamón Guillamón, José Enrique Yuste, Jesús López-Alcolea, Federico Dicenta, Raquel Sánchez-Pérez

**Affiliations:** ^1^ Department of Plant Breeding, Centro de Edafología y Biología Aplicada del Segura- Consejo Superior de Investigaciones Científicas (CEBAS-CSIC), Campus Universitario de Espinardo, Murcia, Spain; ^2^ Metabolomics Platform of CEBAS-CSIC, Campus Universitario de Espinardo, Murcia, Spain

**Keywords:** agrochemicals, endodormancy release, metabolomics, peach, phenylpropanoids, phospholipids, global warming

## Abstract

**Introduction:**

In Mediterranean areas like Spain, global warming has endangered stone fruit production by reducing chill accumulation, leading to significant agronomical and economical losses. To mitigate this issue, agrochemicals have been applied for decades to promote endodormancy release and initiate flowering. However, many of these chemicals have been associated with strong phytotoxicity, resulting in their recent ban. As a result, identifying novel pathways to modulate endodormancy release is critical and essential for developing effective, non-toxic agrobiochemicals.

**Methods:**

In this study, we investigated the effect of two different agrochemicals mixes: 1% Broston^®^ + 5% NitroActive^®^ and 3% Erger^®^ + 5% Activ Erger^®^, sprayed on peach trees during endodormancy over two years, followed by non-target metabolomic analyses on flower buds to identify metabolic changes in treated versus control trees.

**Results and discussion:**

Significant variations were observed in metabolites from the abscisic acid and phenylpropanoid pathways. Notably, six types of phospholipids were identified, with most increasing exclusively in treated samples during endodormancy release. These results were in concomitance with the increased expression of *4-coumarate-CoA ligase 1*, *9-cis-epoxycarotenoid dioxygenase*, and *xanthoxin dehydrogenase* genes. In a subsequent validation experiment performed in the third year, branches treated with phospholipids and cinnamic, caffeic, and abscisic acids advanced endodormancy release by one to two weeks, which represents the first evidence of endodormancy release modulation by the use of these metabolites. Moreover, this study contributes to our understanding of the biochemical mechanisms involved in endodormancy release and highlights the potential of phenylpropanoids, phospholipids, and related compounds as targets for developing sustainable agrochemicals, addressing challenges posed by climate change to *Prunus* spp. cultivation.

## 1 Introduction

Woody perennial plants have the ability to survive winter temperatures through a physiological “hibernation” process named endodormancy (Kovaleski, 2022). However, this stage is not merely a survival strategy, as successful flowering is highly dependent on cold winter temperatures, which directly influence plant productivity (Fadón et al., 2020). Proper endodormancy is crucial for achieving high yields (Luedeling et al., 2009). The transition from endodormancy to the subsequent phase, ecodormancy, is determined by the chilling accumulated in flower buds. Only once a specific chilling accumulation threshold is reached – referred to as chilling requirements (CR) – can trees exit endodormancy. This threshold is variable among species and cultivars (Sánchez-Pérez et al. 2012; Martínez-Gómez et al. 2017). Temperate woody fruit species, such as those in the Rosaceae family – like peach (*Prunus persica* [L.] Batsch), almond (*Prunus dulcis* [Mill.] D.A.Webb), apricot (*Prunus armeniaca* L.), sweet cherry (*Prunus avium* [L.] L.), and plum (*Prunus salicina* Lindl.) – are prime examples of plants dependent on endodormancy. These fruit species are widely cultivated in temperate regions worldwide, with an aggregated production of 47 Mtons across 7.3 Mha (FAOSTAT, 2020).

In recent decades, rising winter temperatures, driven by global warming, have led to inadequate winter chill accumulation, failing to meet the necessary CR for many species (Delgado et al., 2021). This issue is particularly concerning in temperate regions such as the Mediterranean area, where average annual temperatures had increased by 1 to 1.5°C over the past 50 years. Furthermore, projections for 2050 indicate that this warming trend will continue, resulting in a significant reduction in chill accumulation in these regions (Fernandez et al., 2023). Such changes pose a serious threat to the productivity and sustainability of temperate fruit crops, as insufficient chill may disrupt key phenological events like flowering and fruit set, ultimately affecting yield and fruit quality.

To address this challenge, farmers have turned to various agrochemicals to promote endodormancy release and flowering, thereby ensuring a proper annual yield. Over the past few decades, Dormex^®^ has been the most effective agrochemical on the market, contributing to the cultivation of over 1,500 Mtons across approximately 1,100 Mha. However, due its toxicity to humans and the environment, Dormex^®^ has been banned in EU, which in turn has led to an increased demand for novel agrochemicals that facilitate endodormancy release (Sheshadri et al., 2011; Ionescu et al., 2017a; Ionescu et al., 2017b; Guillamón et al., 2022). Among them, compounds such as Erger^®^ + Activ Erger^®^ and Syncron^®^/Broston + NitroActive^®^ are particularly noteworthy, which, like Dormex^®^, are based on high-organic nitrogen solutions (**Table 1**). Despite of their lower toxicity, these alternatives also exhibit lower efficiency than Dormex^®^.

**Table 1:** Composition of the most used agrochemicals worldwide.

Agrochemical	Composition
Dormex^®^	52% Hydrogen cyanamide
Erger^®^	3.1% Ammoniacal nitrogen + 5.8% Nitrate nitrogen + 6.1% Urea nitrogen + 3.3% Calcium
Activ Erger^®^	9% Nitric nitrogen + 6% Ammoniacal nitrogen + 6.5% CaO
Syncron^®^	2% Free amino acids + 0.3% Total nitrogen + 80% Total organic matter
NitroActive^®^	11.5% Nitric nitrogen + 5.5% Ammoniacal nitrogen + 12.3% CaO
Broston^®^	Under trade secret protection (request to the authors)
Bluprins^®^	0.7% Free amino acids + 4% Nitric nitrogen + 4% Ammoniacal nitrogen + 5.5% Organic carbon
Bluact^®^	9% Nitric nitrogen + 6% Ammoniacal nitrogen + 7% CaO
Kiplant HB15^®^	9% Nitric nitrogen + 6% Ammoniacal nitrogen + 6.1% CaO
Kiplant Inducer^®^	6.5% Nitric nitrogen + 3% Ammoniacal nitrogen + 6.1% Urea nitrogen + 6.1% CaO

Studies on apple, peach, and sweet cherry have identified concentration, chill accumulation and cultivar type as critical factors influencing the effectiveness of agrochemical application (Campoy et al., 2011; Rodrigo et al., 2017). Any miscalculation in these parameters could result in an overaccumulation of reactive oxygen species (ROS) in the plant, leading to phytotoxicity, which ultimately may drop annual yield. Therefore, there is a pressing need for reliable endodormancy release biomarkers to ensure the correct application of existing agrochemicals and to support the development of new, environmentally friendly agrobiochemicals that are as efficient as Dormex^®^.

The objective of this work was to identify potential biomarkers for endodormancy release and to develop new agrobiochemicals by investigating the molecular effects of two commercially available agrochemicals, with the aim of enhancing bud dormancy release in peach. Non-target metabolomic analysis was conducted to examine the application effect of Erger^®^ + Activ Erger^®^, and Broston^®^ + NitroActive^®^ on peach flower buds over two consecutive years. As a result, significant metabolites whose concentration either increased or decreased in the flower bud tissues, were tested in a third year, leading to, for the first time, observable advancements in endodormancy release. To the best of our knowledge, this is the first successful validation of metabolites identified through metabolomic analyses as modulators of endodormancy release in a perennial plant like peach. Consequently, this research represents a stepping stone in addressing the negative impacts of global warming on economically important fruit tree species, particularly those cultivated in temperate regions.

## 2 Materials and methods

### 2.1 Plant material

Peach trees of the Carioca cultivar, which is characterized by an intermediate flowering time, were grown in the commercial farm “Agrícola Don Fernando, Frutas Esther, SA” (latitude: 38° 7’ 39.52’’ N, longitude 1° 3’ 16.38’’ W, Campotéjar, Molina de Segura, Murcia, Spain). This is a mild winter region, which climatic data, between 2019-2021, indicating an average annual temperature of 17-18 °C, relative humidity of 55-57%, wind speeds ranging from 1.70 to 1.63 m/s, and average precipitation of 384 mm (Murcia Agrarian Informatic System: SIAM). All the selected trees were over 5 years old and 4 m in height, grafted onto the peach rootstock Rootpac 70^®^, with a planting density of 3 x 5 m.

### 2.2 Determination of chill accumulation

To ensure timely application of the agrochemicals, chill accumulation was monitored on the three years of study by recording hourly temperatures from the meteorological station at Campotéjar. This temperature data was then input into the Dynamic model (Fishman et al., 1987), which is widely regarded as the most accurate tool for measuring chill accumulation in mild-winter regions, such as the Southeast of Spain (Prudencio et al., 2018). Chill accumulation was expressed in chill portions (CP). According to the technicians of Frutas Esther, SA, the average chill requirements (CR) of Carioca in Campotéjar experimental field are approximately 43 CP.

### 2.3 Agrochemical applications and sampling

Trials were conducted when 31 CP were accumulated (representing about 70% of the theoretical CR needed to break endodormancy release). The treatments applied were: 1% Syncron^®^/Broston^®^ + 5% NitroActive^®^ (treatment B, TB) and 3% Erger^®^ + 5% Activ Erger^®^ (treatment E, TE), by using a 10L backpack manual sprayer (2L/whole tree), between 9 and 10 am, at a single time, under low wind conditions. In total, 10 trees per treatment were applied for TB and TE, distributed each treatment in independent rows, separated by 40 m from the trunk, to avoid cross-contamination. Control trees were left unsprayed, following the procedure used by Guillamón et al. (2024).

**Figure 1:**
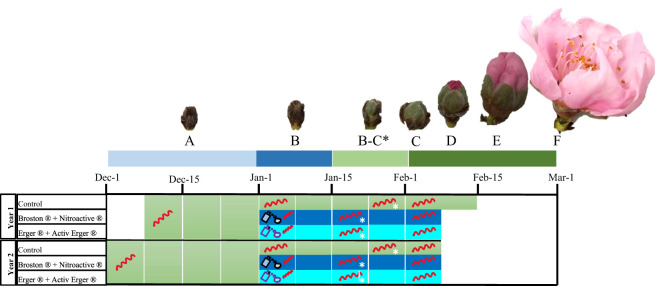
Chart of the treatments applied to peach flower buds. From December to March, samples were analyzed at various stages: deep endodormancy **(A)**, endodormancy **(B)**, endodormancy release **(B, C)** and in ecodormancy **(C-F)**. Treatment application and endodormancy release (**B-C** stage) dates are indicated by a spray icon and an asterisk, respectively. Metabolomic samples were collected weekly throughout the colored periods for all sample groups, while expression analyses were conducted on samples marked with a single-strand mRNA molecule.

For each treatment, three consecutive neighboring trees were selected for sampling and further experimentation, whereas the other 7 trees were used to estimate flowering and ripening times (**Figure 1**). Flower buds (1 g) were collected weekly from deep endodormancy (December 1 and November 30 in years 1 and 2, respectively) to flowering (February 18 and 12 in years 1 and 2, respectively). To standardize flower bud development across the samples, three twigs per tree – each containing both flowers and vegetative buds (mixed twigs) – were randomly selected from the same height in the tree canopy, but from different orientation. In total, 9 twigs were collected per treatment, with an additional 9 from the control trees.

### 2.4 Determination of the dormancy status

From December to mid-February, during the two seasons (2019/2020-2020/2021), three mixed twigs (40 cm long and 5 mm in diameter) were weekly picked from three neighboring trees and placed in a growth chamber under controlled conditions (23 ± 1°C, and 70% relative humidity (RH) during the 16 h of photoperiod; 20 ± 1°C and 70% RH during the dark period) in a 5% sucrose solution with 0.1% of aluminum sulfate (Merck, Darmstadt, Germany). After 10 days in the growth chamber, the phenological stage of the flower buds was evaluated. Endodormancy release was determined when at least 50% of flower buds passed the developmental stage B-C (Baggiolini, 1952) (**Figure 1**), in which A corresponds to fully endodormant buds and C to buds where the calyx can be observed. Once the treatments were performed, treated and untreated samples were immediately transferred from the field to the growth chamber (in about 30 min), to evaluate the dormancy status by calculating the endodormancy release date of both control and treated trees. The first sample of treated trees (and its respective control) was a week after the treatment date.

### 2.5 UHPLC-QToF-MS/MS analysis of flower buds

Three biological replicates, each one consisting of a pool of flower buds (45-55 mg) were ground into fine powder in liquid nitrogen. Subsequently, samples were mixed with 200 µl of CH_3_CN:H_2_O (80:20,*v*/*v*) containing 0.1% HCOOH (Merck), mechanically shaken, sonicated 3 times for 30 s and centrifuged for 10 min at 13,000 x *g*. Glipizide (Merck) at 0.1 µg/ml was added as internal standard. Finally, samples passed through a 0.22 µm filter plate (Merck) by centrifugation (5 min, 1,107 x *g*, 4°C). As this is an untargeted metabolomics analyses, no technical replicates were used, as previously done by Guillamón et al. (2020, 2024).

The UPLC–QToF–MS/MS analyses were done using Waters ACQUITY UPLC I-Class System (Waters Corporation, Milford, MA, USA) coupled to a Bruker Daltonics QToF- MS mass spectrometer [maXis impact Series with a resolution ≥ 55,000 FWHM Bruker Daltonics, Bremen, Germany]. In these analyses ESI was also used for both positive- [ESI (+)] and negative- [ESI (−)] ionization modes.

The column for the UPLC separation was a HSS T3 C18 100 × 2.1 mm with 1.8 µm of size particle (Waters Corporation) at a flow rate of 0.3 mL/min. The weak mobile phase (A) for the separation was H_2_O with a 0.01% of HCOOH (pH ~3.20) (PanReac AppliChem, Barcelona, Spain), while the strong mobile phase (B) was ACN with a 0.01% of HCOOH (J. T. Baker, New Jersey, USA). The gradient started with 10% of B at 0 min, which was progressively increased up to 90% at minute 14; after that, it was held until minute 16 and then decreased to 10% in 10 s, where it remained until minute 18. Nitrogen was used as the desolvation and nebulizing gas with a flux of 9 L/min and a pressure of 2.0 bar. The drying temperature was 200°C and the column temperature was 40°C. The voltage source was 4,000 V for ESI (−) and 4,500 V for ESI (+). The MS experiment was carried out using HR-QTOF-MS, applying 24 eV for ESI (+) and 20 eV for ESI (−) and using broadband collision-induced dissociation (bbCID). MS data were acquired over an m/z range of 45–1,200 Da. A KNAUER Smartline Pump-100 delivered the external calibrant solution with a pressure sensor (KNAUER, Berlin, Germany). The instrument was calibrated externally before each sequence with a 10 mM sodium formate solution. The mixture was prepared by adding 0.5 mL of formic acid and 1.0 mL of 1.0 M sodium hydroxide to an isopropanol/Milli Q water solution (1:1, *v*/*v*).

### 2.6 Data processing and statistical analysis of the metabolomic study

The raw intensity data was acquired using the Profile Analysis 2.1 software (Bruker Daltonics, Germany). In the first analysis, 232 features with a signal-to-noise ratio of 3 for ESI (-) and 3,900 features with a signal-to-noise ratio of 3 for ESI (+) were observed. In the second analysis, 300 features with a signal-to-noise ratio of 3 for ESI (-) and 2,800 features with a signal-to-noise ratio of 3 for ESI (+) were found.

For statistical analysis, MetaboAnalyst 5.0 free software was utilized. Data were logarithmically transformed before applying ‘Pareto scaling’. To identify features exhibiting significant variations between endodormancy and ecodormancy, a Volcano Plot was generated based on *p*-value and fold changes (FC). The threshold for statistical significance was set to a log_2_ (FC) ≥ 2 and a *p*-value ≤ 0.05. Two separate Partial Least‐Square Discriminant Analysis (PLS-DA) models were conducted for each year to detect differences between endodormant/ecodormant buds and control/treated buds. To evaluate the robustness of the models, cross-validation was performed to assess the explained variance and the prediction capability, represented by the R^2^ and Q^2^ scores, respectively.

### 2.7 Metabolite identification

The most significant metabolites were putatively identified by comparing their MS/MS spectra with the data from the following databases: METLIN, Human Metabolome Database (HMDB), and LipidMaps. These metabolites were then grouped according to their biochemical and chemical nature using the KEGG PATHWAY and the ChEBI databases. To assess the accuracy of the putative identifications, we applied the SmartFormula Manually annotation (part of Bruker Daltonik Data Analysis Suite, Bremen, Germany) to assign theoretically elemental compositions to specific m/z values. SmartFormula Manually generates formula suggestions based on accurate mass and isotopic pattern information using the Sigma algorithm, which combines the standard deviations of mass and intensity values for all peaks. The mass tolerance was set to ≤ 3 mDa, the charge to +1, and the H/C element ratio filter was set between 1 and 3. MS/MS spectra were further analyzed by SmartFormula 3D for additional identification insights. To aid in the identification of complex metabolites, such as phospholipids, we analyzed MS/MS spectra of several pure phospholipid standards: 16:0-18:1 phosphatidyl serine (PS), 16:0-18:1 phosphatidic acid (PA), 16:0-18:1 phosphatidyl choline (PC), 16:0-18:1 phosphatidyl ethanolamine (PE), and 16:0-18:1 phosphatidylglycerol (PG). This allowed us to assign the correct polar head ions of each phospholipid: PS head (m/z=290.1336), PA head (m/z=187.0673), PC head (m/z=289.2173), PE head (m/z=230.1373), and PG head (m/z=277.1373).

Finally, we assessed the statistical correlation between the significant compounds that varied between endodormancy and endodormancy release across all study groups. Pearson’s *r* correlation coefficient was used to measure the metric similarity distance between metabolites and the different treatments applied, and a heatmap was generated for changes in metabolites related to control and treated samples.

### 2.8 Enrichment and pathway analysis

The metabolites identified in the two-year study were used to conduct an enrichment analysis, aiming to pinpoint metabolic groups that were over-represented during endodormancy release in both treated and control trees. Additionally, we performed a pathway analysis to examine the key metabolic pathways that changed during endodormancy release across all samples. All analyses were carried out using MetaboAnalyst 5.0 software.

### 2.9 RNA extraction for the expression analysis

For the expression analyses, we consulted the KEGG PATHWAY database to identify genes directly involved in the biosynthesis or degradation of the significant metabolites previously identified (**Table 2**). In addition, we included genes of interest from the literature found on other fruit species such as *4-COUMARATE-CoA LIGASE 1* and *HOMEOBOX-LEUCINE ZIPPER PROTEIN ATHB-12*.

**Table 2:** Primers sequence of the genes analyzed by RT-qPCR.

Gene	Forward primer	Reverse primer	Amplicon size (pb)	Melting temperature (°C)	Efficience (%)	R^2^
*4-Coumarate ligase 1 (4CL1)*	GTTCAGCAGGAGCCACTT	ACAAGAATGGATGGCTTCA	123	F: 59.10; R: 56.00	90.38	0.97
*Xanthoxin dehydrogenase (ABA2)*	GAGTTGGGAGTGCATGGCAT	CAGCCGTCATATCCATTTTCCC	111	F: 62.94; R: 61.04	86.87	0.99
*Glutathionylspermidine synthase (GSS)*	TCCTCAGCCCTCCCAACTTC	CGGTTGGTTGCTGGACATTTT	118	F: 61.40; R: 68.84	84.94	0.99
*CYP707A1*	TCCCATCTGGTTCAAGCCAACC	TGAAGGCACGGAGGACAAG	120	F: 60.58; R: 60.11	81.33	0.98
*CYP707A2*	ATGTTTGCCGCTCAGGAC	TGGCCTTCTGCTCAATCTGG	105	F: 60.61; 60.22	103.88	0.99
*CYP707A4*	AGGTTGTGTTGGAGAGTTTGAG	GGCATCACCTTCCAACCTTT	114	F: 60.29; R: 60.29	84.05	0.99
*the 60S ribosomal protein (60S)*	ATACCAGTTGAAGGATCGT	GGAATATGAGTTGCTAAGAAGG	99	F: 55.63; R: 54.77	99.06	0.99
*Outer envelope pore protein 16 chloroplastic (OEP16)*	CCACGGACCCTTTCTAAA	TGAGCACACTTTGAAGAAG	105	F: 55.45; R: 55.44	99.14	0.99
*Elongation factor 1-alpha (TEF2)*	GGTGTGACGATGAAGAGTGATG	TGAAGGAGAGGGAAGGTGAAAG	129	F: 57.00; R: 57.00	106.34	0.99

RNA was extracted from flower buds of three biological replicates, which were derived from the same pool used for the metabolomic analyses (**Figure 1**). Sampling occurred at four different stages during endodormancy and ecodormancy, as outlined by Baggiolini (1952). These stages included: deep endodormancy (A: first sampling date, at least one month before the treatment), treatment application date (B: one day after treatment, 1 dat), endodormancy release (B-C: 14 dat for treated samples and 21 dat for control samples), and late ecodormancy (C: final sampling date, 35 dat for treated and 42 dat for control samples).

RNA extraction followed the protocol provided by the *NucleoSpin RNA Plant and Fungi* kit (MACHEREY-NAGEL, Düren, Germany). After the extraction, RNA quality was measured by calculating the A_260 nm_/A_280 nm_ and A_260 nm_/A_230 nm_ ratios using a *Nanodrop One* (Thermo Fisher Scientific, Watlham, MA, USA). With this quality control, RNA was purified of genomic DNA and other contaminants using the *DNA-free™, DNase Treatment and Removal Reagents* kit (Thermo Fisher Scientific, Watlham, MA, USA) and the *RNeasy^®^ PowerClean^®^ Pro Clean Kit* (QIAGEN, Venlo, Netherlands).

### 2.10 qRT-PCR analysis

Quantitative reverse transcription PCR (qRT-PCR) was performed in 10 μL reactions using the qPCRBIO SyGreen 1-Step Go Hi-ROX kit (BIOSYSTEMS, London, UK) according to the manufacturer´s instructions. For each sample, 5 ng of RNA was used to quantify the expression of the selected candidate genes. The following PCR protocol was used: 10 min at 45°C, 2 min at 95°C, [5 s at 95°C, 30 s at 60°C, 1x plate read] *x* 40 cycles. A melting curve was generated for each reaction, which included on 15 s at 95°C, 1 min at 60°C and 15 s at 95°C. qRT-PCR was performed using the *PCR StepOnePlus* system (Thermo Fisher Scientific).

Primers for the candidate genes identified in the metabolomic analysis were designed using *CLC Genomic Workbench 5* (QIAGEN). Only primers with an efficiency greater than 80% were used for qRT-PCRs, and primer efficiency was confirmed by the Standard Curve method (**Table 2**).

Relative gene expression from qRT-PCR data was calculated considering the primer efficiency and normalized against the following housekeeping genes: *60S RIBOSOMAL PROTEIN GENE* (*60S*), *OUTER ENVELOPE PORE PROTEIN 16 CHLOROPLASTIC GENE* (*OEP16*), and *TRANSLATION ELONGATION FACTOR 1-ALPHA* (*TEF2*) (**Table 2**). Subsequently, statistical analyses were performed using Tukey’s test in *InfoStat v.2018* (InfoStat, Córdoba, Argentina) to assess significant differences between treated and control samples.

### 2.11 Validation of significant metabolites detected in the first two years

After identifying significant metabolites following agrochemical application, we wanted to assess their effects when externally applied to peach flower buds. In the third year of the study, when 70% of the CR had been met, groups of five 40-cm mixed twigs were collected weekly, for three consecutive weeks, from control trees in the field. Upon arrival at the laboratory, the twigs were sprayed with a selection of commercially available metabolites, including caffeic acid, p-coumaric acid, cinnamic acid, L-phenylalanine (L-Phe), spermidine, abscisic acid and phospholipids (Merck). A set of 5-twigs was retained as untreated control. All metabolites were applied in a 5% aqueous solution, except for caffeic acid, which was applied in a saturated solution. To enhance the solubility of phospholipids, 0.5% Triton™ X-100 (Merck) was incorporated in the solution. The metabolites were applied using a 50 mL spray bottle until drop-off. After drying in a fume cupboard, the twigs were transferred to a growth chamber to evaluate the endodormancy release in both treated and control samples, as previously described in Section 2.4.

## 3 Results

### 3.1 Chill accumulation, endodormancy release and flowering

**Figure 2:**
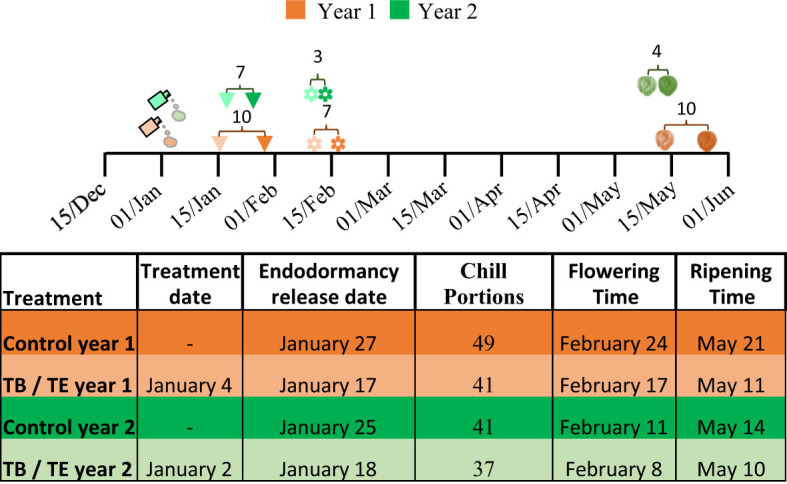
Experimental setup for the two-year study. The timeline table presents the complete experimental schedule, with key events indicated as follows: treatment dates are marked by a spray icon, endodormancy release by a triangle, flowering time by a flower icon, and ripening time by a peach icon. Light orange represents the treatments Broston^®^ + NitroActive^®^ (TB) and Erger^®^ + Activ Erger^®^ (TE) in year 1, while light green represents the treatments in year 2. Dark colors correspond to the respective control periods. Numbers in the square brackets indicate the days advanced in endodormancy release, flowering and ripening times between treated and control trees, as detailed in the table below.

In the first year of the study, treated trees released from endodormancy after accumulating 41 CP on January 17, whereas controls required 49 CP, which occurred on January 27 (**Figure 2**). In the second year, which was warmer (average temperature of 17.93°C in year 1 versus 18.15°C in year 2), treated trees released from endodormancy after accumulating only 37 CP (January 18), while controls accumulated 41 CP (January 25). These results demonstrate that endodormancy release occurred earlier (seven to 10 days) in treated trees, regardless of the agrochemical used or the presence of warmer/colder conditions. With respect to flowering time, treated trees flowered one week earlier than controls in the first year and three days earlier in the second year (**Figure 2**). This trend was also observed in ripening times, where treated trees were harvested between ten (year 1) and four (year 2) days earlier than controls (**Figure 2**).

### 3.2 Metabolomic profiling

**Figure 3:**
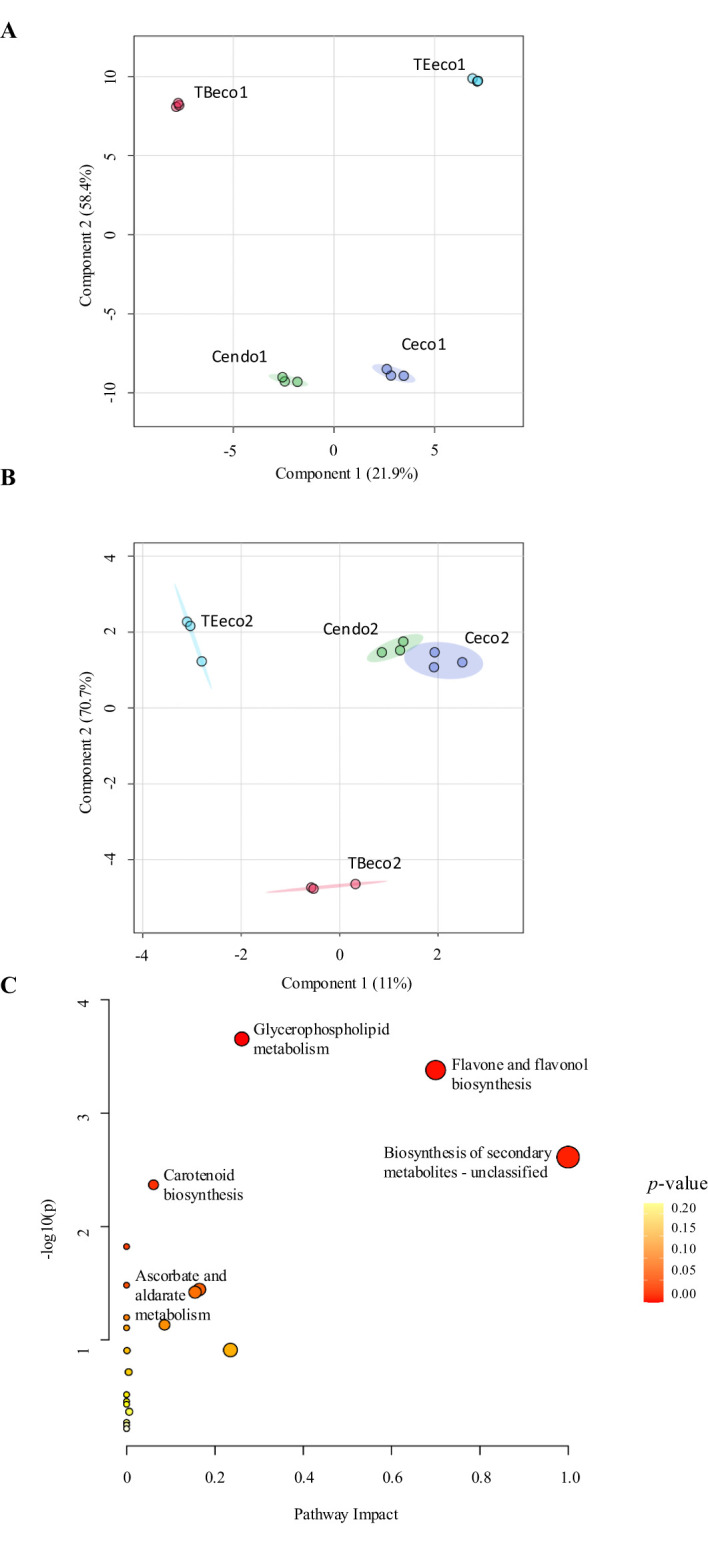
Variations in the metabolic profiling. **(A)** PLS-DA model showing differences between control and treated samples in the first year of the study. **(B)** PLS-DA model from the second year of the study. Cendo: endodormant control samples, Ceco, ecodormancy release control samples; TBeco, endodormancy release samples treated with Broston^®^ + NitroActive^®^; TEeco, endodormancy release samples treated with Erger^®^ + Activ Erger^®^. **(C)** Pathway analysis of metabolites that significantly varied during the two years of study. Node size represents the impact of the pathway, while node color (ranging from white to red) indicates the significance (-log_10_[*p*-value]) of each pathway.

The raw data obtained from the non-target metabolomic analysis were used to perform two PLS-DA models, aiming to observe the differences between control and treated samples across the two years of study. The models showed a clear separation between control samples in endodormancy (Cendo) and in ecodormancy (Ceco) trees, as well as between both Cendo and the treated trees in ecodormancy (Teco) and Ceco versus Teco trees, during both years of study (**Figures 3A, B**). Additionally, the models showed a large distance between Cendo, Ceco and Teco in the first year (**Figure 3A**), whereas in the second year, Cendo and Ceco groups were much closer (**Figure 3B**). The separation between endodormant and ecodormant flower buds was mainly attributed to the significant variation in 100 and 52 metabolites in the first and second year, respectively (**Supplementary Tables S1**, **S2**).

### 3.3 Enrichment and pathway analysis

The enrichment analysis performed on the metabolites that significantly varied during endodormancy release revealed that triterpenoids, the phospholipid species PE, glycerophosphoserines and PA were significantly enriched (**Supplementary Figure S1**). Regarding the pathway analysis, “Glycerophospholipid metabolism”, “Flavone” and “Flavonol biosynthesis”, “Secondary metabolites” and “Carotenoid biosynthesis” pathways showed the lowest *p*-value and the highest pathway impact, being the most relevant pathways across the two years of study (**Figure 3C**).

### 3.4 Phospholipids

**Figure 4:**
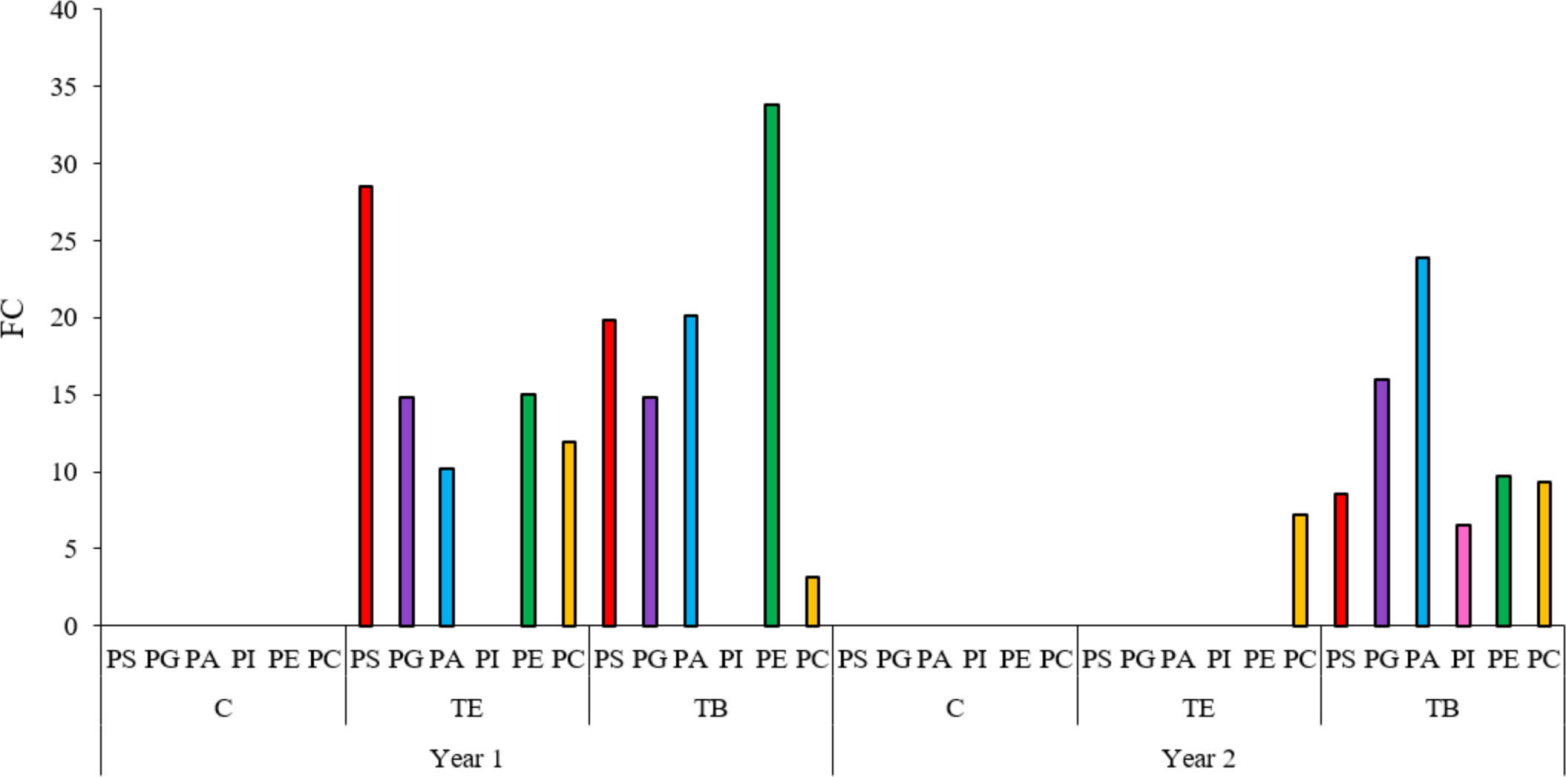
Phospholipid increase during endodormancy release. Bar plot showing the sum of fold changes (FC) for each phospholipid species when comparing deep endodormancy (A stage) and endodormancy release (B-C stage) in treated samples. Colored bars represent different phospholipid species: red for phosphatidyl serines (PS), purple for phosphatidyl glycerols (PG), blue for phosphatidic acids (PA), pink for phosphatidyl inositols (PI), green for phosphatidyl ethanolamines (PE), and yellow for phosphatidyl cholines (PC). Treatment B (TB) involved 1% Broston^®^ + 5% NitroActive^®^ and treatment E (TE) was done with 3% Erger^®^ + 5% Activ Erger^®^. Significant differences in phospholipid variation were determined using a Volcano plot (FC > 2 and *p*-value < 0.05). The specific FC and *p*-value for each phospholipid is shown in **Supplementary Tables S1** and **S2**.

As previously presented, among all the metabolic pathways that showed significant variations during endodormancy release across the different years and groups, glycerophospholipid metabolism – in which phospholipids are biosynthesized – was the most significant pathway during endodormancy release. In general, phospholipids exhibited a significant increase, measured as fold change (FC), during endodormancy release in the two years of study. A total of six types of phospholipids were identified: PS, PG, PA, phosphatidyl inositols (PI), PE, and PC. For each type, up to 15 different subtypes were identified (see **Supplementary Tables S1, S2**). In the first year, most identified phospholipids significantly increased in both treated groups, while remained unchanged in controls (**Figure 4**). In the second year, all the identified phospholipids increased in TB samples, and only PC increased in TE samples. PEs were the phospholipid species showing the highest FC in both treated samples in the first year, while PAs showed the highest FC in the TB-treated samples (in the second year only). This variation was particularly pronounced in treated samples across both years of study (**Figure 4**).

### 3.5 Biosynthesis of secondary metabolism: phenylpropanoids

**Figure 5:**
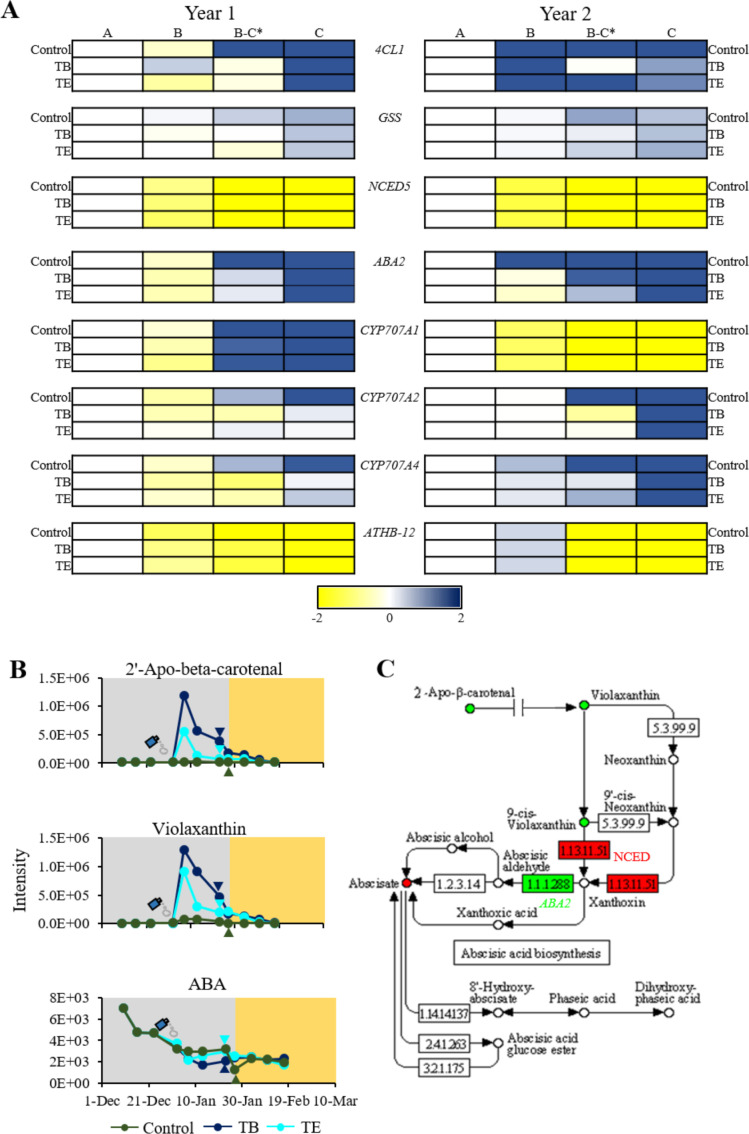
Metabolic and genetic variation observed during endodormancy release **(A)**. Heatmap of relative expression levels for *4CL1, GSS, NCED5, ABA2, CYP707A1, CYP707A2*, *CYP707A4*, and *ATBH-12* during the transition from endodormancy to ecodormancy. Stages include: A (deep endodormancy), B (medium endodormancy), B-C (endodormancy release) and C (ecodormancy). Genes shown are: *4-Coumarate ligase 1* (*4CL1*), *Glutathionylspermidine synthase* (*GSS*), *Xanthoxin dehydrogenase* (*ABA2*; 1.1.1.288), *9-cis-Epoxycarotenoid dioxygenase* (*NCED5*; 1.1.1.51), *CYTOCHROME P450 707A1* (*CYP707A1*), *CYTOCHROME P450 707A2* (*CYP707A2*), *CYTOCHROME P450 707A4* (*CYP707A4*), and *HomeoBox-Leucine Zipper Protein ATHB-12* (*ATBH-12*). Samples correspond to control, treatment B (TB) (1% Broston^®^ + 5% NitroActive^®^), and treatment E (TE) (3% Erger^®^ + 5% Activ Erger^®^). The endodormancy release date is marked with an asterisk. **(B)** Variation in metabolites from the ABA biosynthetic pathway during year 1. Agrochemical application dates are marked with a spray icon, and the endodormancy release date is indicated by with a colored triangle. Dark green represents control samples, royal blue represents TE samples, and dark blue, TB samples. The background color highlights the endodormancy period (grey) and the ecodormancy period (yellow) for the control group. **(C)** Metabolic and genetic variations in the ABA biosynthetic pathway. Red color indicates a decrease in expression or concentration, while green indicates an increase.

Phenylpropanoids are by-products of the metabolism of L-Tyr and L-Phe. In our study, we identified a total of five phenylpropanoids, all by-products of p-coumaric acid, that significantly varied during endodormancy release across all groups and years. These included p-coumaric acid, trans-3-feruloylcorosolic acid, 3-O-trans-feruloyleuscaphic acid, 1,28-dicaffeoyloctacosanediol and p-coumaric acid. All of these metabolites exhibited a consistent trend, with significant increases (FC > 2) during endodormancy release in both study years. This increase was particularly pronounced in treated samples (**Supplementary Figures S4**, **S5**, **S7**, **S8**). Moreover, given the apparent role of phenylpropanoids in endodormancy release (Conrad et al., 2019), the relative expression of *4-coumarate ligase 1 (4CL1)*, gene responsible of the biosynthesis of most of phenylpropanoids, was examined in the samples from both years (**Figure 5A**). In all groups, the *4CL1* gene remained almost unexpressed during endodormancy, with a pronounced peak in expression following endodormancy release (**Figure 5A**).

### 3.6 Antioxidant species: flavonoids and ascorbic acid metabolism

The number of flavonoids that significantly varied in treated samples was markedly higher than the observed in controls (**Supplementary Tables S1**, **S2**). Flavonols like kaempferol-3-glucosyl-(1->3)(4’’’-acetylrhamnosyl)(1->6)-galactoside, quercetin 3-(6’’’-acetylglucosyl)(1->4)-rhamnoside and the flavone tanariflavanone A, among others, exhibited a significant increase during the endodormancy release in the second year of study. On the other hand, ascorbic acid displayed a significant increase in both treated samples during the endodormancy release of the second year (**Supplementary Figures S7**, **S8**). In addition, the relative gene expression of the *glutathionylspermidine synthase* (*GSS*), involved in ascorbic acid biosynthesis, was analyzed in all the samples. In both years, an upregulation during both endodormancy release and ecodormancy was observed across all groups (**Figure 5A**).

### 3.7 Abscisic acid metabolism

The expression analysis of several genes involved in ABA biosynthesis, catabolism, and response revealed significant upregulation and downregulation during endodormancy release. In terms of biosynthesis, the *XANTHOXIN DEHYDROGENASE* gene (*ABA2*) exhibited a significant increase during endodormancy release in both years of study, whereas the *9-CIS-EPOXYCAROTENOID DIOXYGENASE* gene (*NCED5*) gene showed a marked decrease during this period (**Figure 5A**). In terms of ABA catabolism, the genes encoding cytochromes P450s, such as *CYP707A1, CYP707A2* and *CYP707A4*, were significantly upregulated during endodormancy release in both years, except for *CYP707A1*, which was downregulated in the second year (**Figure 5A**). With regard to ABA response, *HOMEOBOX-LEUCINE ZIPPER PROTEIN ATHB-12* (*ATBH-12*) displayed a significant decrease during the first and second year of study (**Figure 5A**).

Additionally, we observed that several ABA precursors, such as 2’-apo-beta-carotenal and violaxanthin, among others, showed a significant increase in samples from both the first and second year (**Supplementary Tables S1**, **S2**). This increase was notably higher in treated samples compared to controls samples. In contrast, ABA levels exhibited a marked decrease during endodormancy release in all samples from the first year, with minimal variation observed between treated and controls, and no detection in the second year, the warmer one (**Figure 5B**). A summary of the metabolic and gene expression data related to ABA is represented in **Figure 5C**.

### 3.8 Triterpenes

Terpenes are metabolites derived from isoprene metabolism. Triterpenes showed significant increase in treated trees across all years, while remained largely unchanged in control trees (**Supplementary Figures S4**, **S5**, **S7**, **S8**). Some isoforms of ganoderic acid also increased in treated samples during the endodormancy release in both the first and second year of study (**Supplementary Tables S1**, **S2**). In the second year, additional triterpenoids such as tsugaric acid A21-glucosyl ester and tyromycic acid, displayed a similar trend to ganoderic acid, with significant increases during endodormancy release (FC > 2) (**Supplementary Table S2**).

### 3.9 Validation of significant metabolites

**Figure 6:**
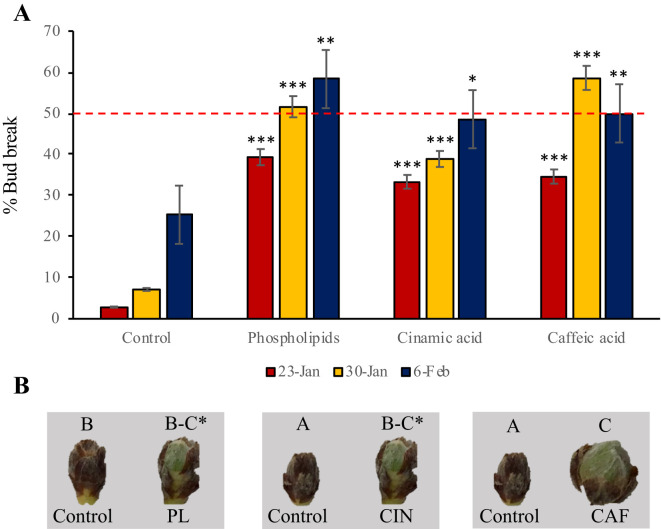
Validation of the results observed in the non-target metabolomic analysis. **(A)** Mean percentage of bud break (% of flower buds in the phenological state B-C) in control and treated branches with pure metabolites after 10 days in the growth chamber. The metabolites applied that influenced the endodormancy release date compared to control samples were phospholipids, cinnamic acid and caffeic acid. The red dashed line indicates the endodormancy release threshold, which corresponds to the point at which 50% of flower buds are in stage B-C. Colored bars represent three experimental dates: red for January 23, yellow for January 30, and dark blue for February 6. Asterisks indicate the level of statistical significance, calculated by a *t*-test relative to the control: one asterisk for a *p*-value of 0.05, two for 0.01, and three for 0.005. **(B)** Comparison of flower buds control and treated branches with phospholipids (PL), cinnamic acid (CIN), and caffeic acid (CAF). The photos were taken on February 16^th^, with all images captured at the same magnification. Phenological stages are labeled at the top of each flower bud. The endodormancy release date is marked on the timeline with an asterisk.

To validate the results from the non-target metabolomic analysis, we applied seven pure standards from the pathways mentioned previously identified as significant in the endodormancy release process. The applied metabolites were selected based on: position within their biosynthetic pathways, their cost and commercial availability. These compounds included the phenylpropanoids caffeic, cinnamic and coumaric acid; L-Phe, spermidine (a precursor of ascorbic acid), and pure phospholipids. Among these, phospholipids, cinnamic and caffeic acid induced an advancement of one to two weeks compared to the control, with caffeic acid and phospholipids having the most pronounced effect on endodormancy release (**Figure 6**). The rest of metabolites did not produce any variation with respect to the control of endodormancy release.

## 4 Discussion

### 4.1 Advance of endodormancy release

In this work, we examined the effects of various agrochemicals on the metabolism of peach flower buds to promote endodormancy release. Previous research has demonstrated the effectiveness of endodormancy release promoters in both tropical and temperate regions (Guillamón et al., 2022; Ionescu et al., 2017a, 2017b; Del Cueto et al., 2017; Rodrigo et al., 2017). In apple (*Malus domestica* Borkh), studies have shown that the timing and concentration of agrochemical application significantly impacts the advancement of endodormancy release (Abreu et al., 2018). Similarly, a recent study on apricot showed that Erger^®^ + Activ Erger^®^, or Broston^®^ + NitroActive^®^ treatments advanced endodormancy release by seven days in a two-year study (Guillamón et al., 2024). In our work, treated trees exhibited an advancement in endodormancy release, regardless of the agrochemical treatment, year or the environmental conditions. This result is consistent with observations in sweet cherry, where high-nitrogen content treatments induced earlier flowering by three to four days compared to control trees (Ferreira et al., 2019; Rodrigo et al., 2017).

### 4.2 Metabolomic profiling

Our findings align with previous works on sweet cherry and kiwi, where agrochemical treatments induced significant variations at the gene expression and cellular levels (Hoeberichts et al., 2017; Ionescu et al., 2017a; Del Cueto et al., 2017). Additionally, recent findings in apricot identified flavone and flavonol biosynthesis, glycerophospholipid metabolism, and secondary metabolites biosynthetic pathways as key processes affected by the Erger^®^ + Activ Erger^®^, or Broston^®^ + NitroActive^®^ treatments (Guillamón et al., 2024).

### 4.3 Phospholipid increments in treated samples

Our analysis revealed that most significant changes in metabolites during endodormancy release were within the phospholipid metabolic group. Earlier studies in peach indicated that the plasma membrane is a crucial cellular component during endodormancy release, with an increase in membrane fluidity playing a vital role in this process (Portrat et al., 1995). Previous research on thidiazuron application to apple flower buds noted significant variations in fatty acids and sterols in apple flower buds, although phospholipid rearrangements was not reported (Wang et al., 1988). More recent studies have highlighted the role of organic phosphorus (P) storage in leaf buds to meet the P demand during and after endodormancy release (Netzer et al., 2018). Additionally, the increase in phospholipid species was linked to an enhancement in plasma membrane fluidity (Beauvieux et al., 2018). In apricot, phospholipid content was also increased significantly during endodormancy release in treated trees compared to controls (Guillamón et al., 2024). Specifically, PA and PE were the two most prevalent phospholipid species in the TB-treated samples during the first year, while PS was more prominent in the second year. In our analysis, PC was the only phospholipid species that significantly increased in all treated groups across both years, with PE showing the highest increase in the first year and PA in the second year. We therefore propose that the accumulation of phospholipids in our samples may be a mechanism for storing the P necessary for endodormancy release and subsequent processes. However, further studies are needed to confirm this hypothesis.

In Arabidopsis, key genes involved in the promotion or repression of flowering present a phosphatidylethanolamine (PE)-binding domain (Colin and Jaillais, 2020), such as *FLOWERING LOCUS* (*FT*), *TERMINAL FLOWER* (*TFL*), among others. Interestingly, the expression of both *FT* and *TFL* follows a circadian rhythm, *i.e*. *FT* expression increases during the day, while *TFL* expression rises at night (Rinne et al., 2011). In addition, the saturation level of phospholipids decreases at night, which could be critical for the specific binding of *FT* to PEs during daytime (Nakamura et al., 2014). Furthermore, *FT* has been shown to possess a selective binding domain for PC, which specifically binds to saturated PCs during the day (Nakamura et al., 2014). This could suggest that the increase in PEs, PCs and PC by-products observed in our study during endodormancy release may facilitate flowering by interacting with an *FT* homolog in peach. Moreover, the significant increase in PEs and PCs suggests a key role of phospholipid metabolism in the modulation of endodormancy release. To the best of our knowledge, this is the first time that external application of phospholipids has been done in peach to advance endodormancy release with a successful result.

### 4.4 Phenylpropanoid rises in treated samples

Phenylpropanoids, by-products of L-Tyr and L-Phe metabolism, are well documented in their involvement in endodormancy release in *Prunus* spp (Conrad et al., 2019; Guillamón et al., 2020; Prudencio et al., 2020). In both almond and apricot, the expression of *4-COUMARATE: CoA LIGASE-LIKE 1* and *7* (*4CL1* and *4CL7*) increased during endodormancy release (Conrad et al., 2019; Prudencio et al., 2020; Guillamón et al., 2024). This is consistent with our findings in peach, where the expression of *4CL1* was significantly increased during endodormancy release in both the first and second year of study (**Figure 5A**). This increase is closely linked to stress responses in many species. For example, in apricot, phenylpropanoids accumulate during late endodormancy and its release, likely in response to reduced ROS levels during this period. Their levels then decline as sepals and petals emerge, suggesting a role in the reactivation of flower buds, as indicated by the phenological changes in bud development during endodormancy (Conrad et al., 2019; Guillamón et al., 2024). To further validate these findings, we applied cinnamic and caffeic acids to mixed twigs in the laboratory, which advanced endodormancy release by one and two weeks, respectively. While the exact mechanisms by which these compounds modulate endodormancy release are not yet fully clarified, a recent study on Japanese apricot (*Prunus mume* [Siebold] Siebold & Zucc.), demonstrated that miRNAs target several genes involved in the regulation (biosynthesis or catabolism) of the phenylpropanoid biosynthesis, modulating the accumulation of these species (Zhang et al., 2022). This previous study supports our findings, confirming the implication of phenylpropanoid biosynthetic pathway in endodormancy release, and suggesting that these compounds could serve as biomarkers for the endodormancy release and potential targets for its modulation.

### 4.5 Reductive species: flavonoids and ascorbic metabolism

Studies in Japanese pear (*Pyrus pyrifolia* (Burm.f.) Nakai) and sweet cherry have shown a rise in ROS levels during endodormancy, followed by a decrease during endodormancy release (Baldermann et al., 2018; Takemura et al., 2015). Our data showed a reduction in dehydroascorbate levels and a simultaneous increase in ascorbic acid during endodormancy release in treated samples. This is consistent with findings in almond, where ascorbic acid levels increased during endodormancy release (Guillamón et al., 2020), and suggests ascorbic acid as a potential biomarker for endodormancy release in *Prunus* (Guillamón et al., 2020, 2024). Additionally, we believe that the accumulation of ascorbic acid observed in our samples, coupled with the upregulation of *GSS*, indicates that the biosynthesis of ascorbic acid and its role in reducing ROS levels is tightly linked to the endodormancy release process.

Flavonoids are antioxidant compounds derived from the metabolism of benzopyrone (Kesarkar et al., 2009). Several subgroups, including flavones, flavonols, and anthocyanins, have been identified for their role in promoting endodormancy release (Michailidis et al., 2018). A metabolomic study in peony tree (*Paeonia × suffruticosa* Andrews), revealed an accumulation of flavonoids prior to endodormancy release (Zhang et al., 2020). Likewise, studies in almond and apricot have demonstrated that flavonoid concentrations increase during and after endodormancy release (Guillamón et al., 2020, 2024). This suggests that the accumulation of flavonoids, along with other antioxidant species, may be essential for releasing endodormancy, likely through the reduction of ROS levels in flower buds.

### 4.6 Rearrangements in ABA metabolism

ABA is a key regulator of endodormancy, its levels decreasing during endodormancy release (Chmielewski et al., 2018; Rodriguez et al., 1994). In grapevine, a reduction in ABA is necessary for endodormancy release (Zheng et al., 2018). Furthermore, treatment with Dormex^®^ in grapevine has been shown to induce both an up- and downregulation of genes involved in ABA biosynthesis during endodormancy release (Ophir et al., 2009). In apple, the drop in ABA during endodormancy release is primarily driven by the inhibition of the *NCED1* gene by miR159 family (Garighan et al., 2021). This finding is consistent with our results, in which the decrease in ABA was associated with the downregulation of the *NCED5* gene in the second year. Conversely, the *ABA2* gene, exhibited the opposite trend to *NCED5*, increasing its expression during endodormancy release.

In sweet cherry, differences in the chill accumulation between years led to significant variations in gene expression related to endodormancy and endodormancy release (Rothkegel et al., 2020). This variability highlights the importance of conducting multi-year studies, as climatic conditions can influence gene expression patterns. Indeed, in our study, ABA levels were no detected in the second year (the warmer one) in all the samples, indicating that the content of this hormone could be more affected by the climate than other factors.

Regarding ABA response, the gene *ATBH-12* showed a significant downregulation, consistent with previous findings in almond during endodormancy release (Prudencio et al., 2020).

Corroborating other reports, by Guillamón et al., 2020; Fadón et al., 2020, and Zheng et al., 2018, during endodormancy release phenylpropanoids, flavonoids, ascorbic acid, and gibberellins increased alongside a decline in ABA during both years of study.

### 4.7 Terpenes increase in treated samples

Terpenes and terpenoids are aromatic compounds found in many plants, particularly in flowers, where they play key roles in attracting pollinators, deterring herbivores, and protecting plants from environmental stresses. These compounds are involved in the plant’s metabolic processes, enhancing their ability to flower and reproduce. They are produced primarily through the mevalonate and methylerythritol phosphate (MEP) pathways. The presence of specific volatiles terpenes like limonene, pinene, or linalool can vary among species and influence the plant’s scent, which is crucial for pollination success (Degenhard et al., 2009). However, the role of terpenes in endodormancy release has rarely been studied in any species. In apricot, triterpens such as violaxanthin and 2’-apo-beta-carotenal raised following the application of agrochemicals to promote endodormancy release (Guillamón et al., 2024). Similarly, in our study, triterpene levels rose during endodormancy release in all the treated samples, which is consistent with previous research in wintersweet (*Chimonnathus praecox*), where genes involved in terpene biosynthesis were upregulated during endodormancy release (Shang et al., 2020). In Arabidopsis, the sesquiterpene trans-caryophyllene has been linked to oxidative stress and water regulation (Araniti et al., 2017). To the best of our knowledge, this is the first study to associate terpenes with endodormancy release in peach, marking a novel finding in the field. Since most of this type of compounds are volatiles, future work should focus on detection of volatile compounds by GC-MS, in order to deep in the role of terpenes in endodormancy release and flowering time in *Prunus* spp.

## 5 Conclusions

In this study, two types of agrochemicals were applied to endodormant flower buds to modulate endodormancy release, resulting in significant variations in metabolic patterns including phospholipids, phenylpropanoids, flavonoids, ascorbic and abscisic acids and terpenes. This emphasize the complex metabolic reprogramming associated with endodormancy release. Because of these facts, we propose ABA, ascorbic and coumaric acid as candidate biomarkers for monitoring endodormancy release in *Prunus* species. Moreover, the successful application of phospholipids and cinnamic and caffeic acids on advancing endodormancy release highlights the potential of these compounds for future development of environmentally friendly agrochemicals in a context of climate change.

## Data Availability

The original contributions presented in the study are included in the article/**Supplementary Material**, further inquiries can be directed to the corresponding author/s.
