# Supplementary material for: Unraveling metabolic shifts in peach under agrochemical treatments during flower bud endodormancy in the context of global warming
Source: Front Plant Sci. 2025 Mar 19;16:1502436. doi: 10.3389/fpls.2025.1502436 (PMC11962625; doi:10.3389/fpls.2025.1502436)
Supplement: Supplementary file 1 [file Image1.pdf]

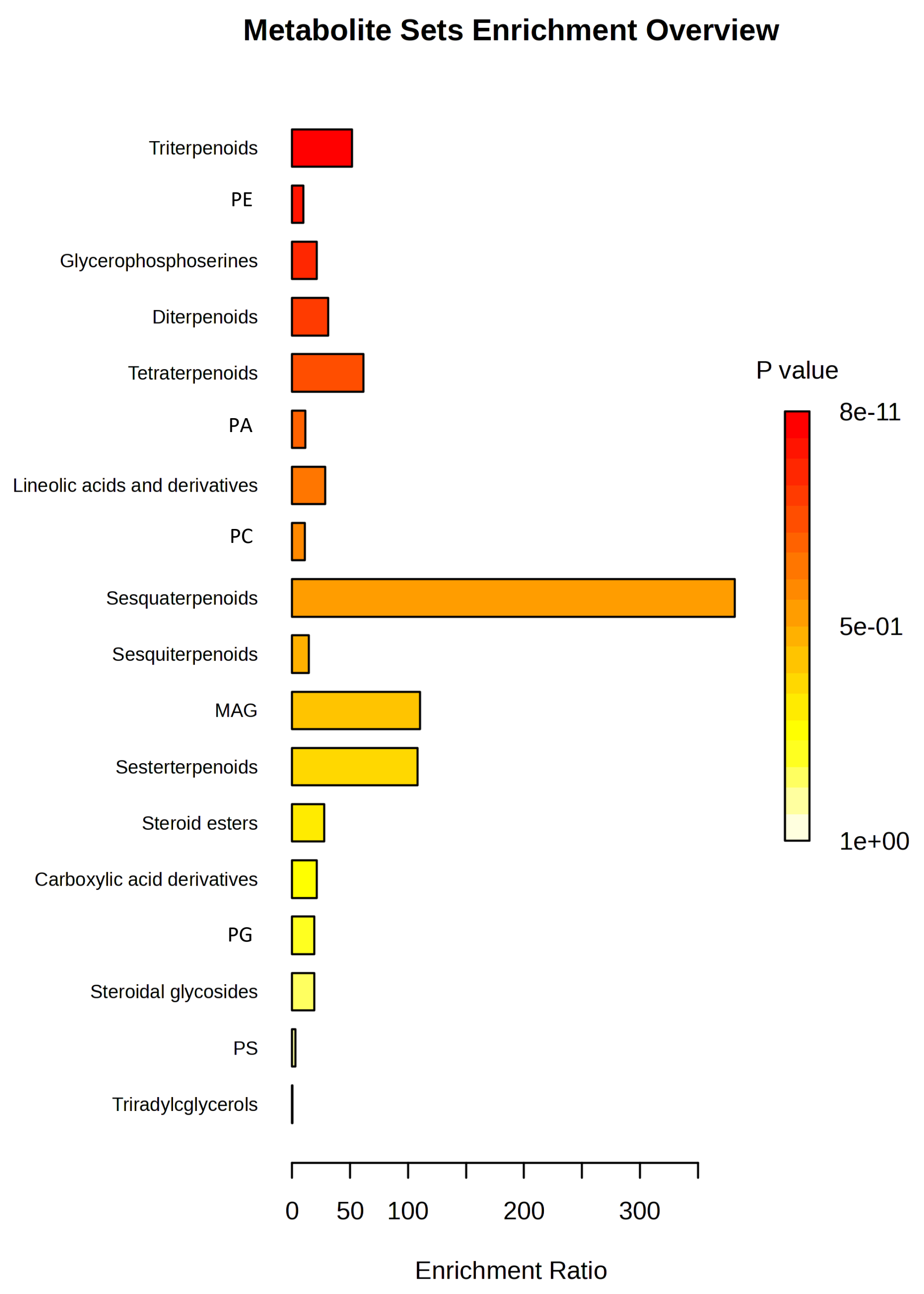

Supplementary figure S1. Enrichment analysis of the metabolic groups from the two years of study. PE: phosphatidyl ethanolamines, PA: phosphatidic acids, PC: phosphatidyl cholines, MAG: monophosphatidyl glycerols, PG: phosphatidyl glycerols, and PS: phosphatidyl serines.

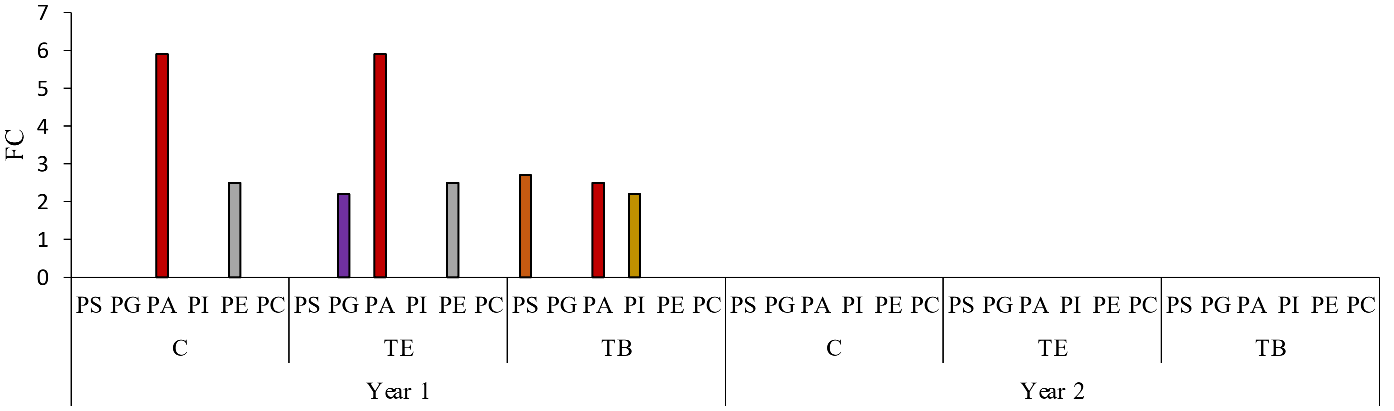

Supplementary figure S2. Phospholipid decrease during endodormancy release. Chart of the accumulated FC of each phospholipid group in control (C), treatment B (TB) (1% Broston® + 5% NitroActive®), and treatment E (TE) (3% Erger® + 5% Activ Erger®) samples. Colored bars indicates the group: phosphatidyl serines (PS, orange), phosphatidylglycerols (PG, purple), phosphatidic acids (PA, red), phosphatidyl inositols (PI, yellow) and phosphatidyl ethanolamines (PE, grey).

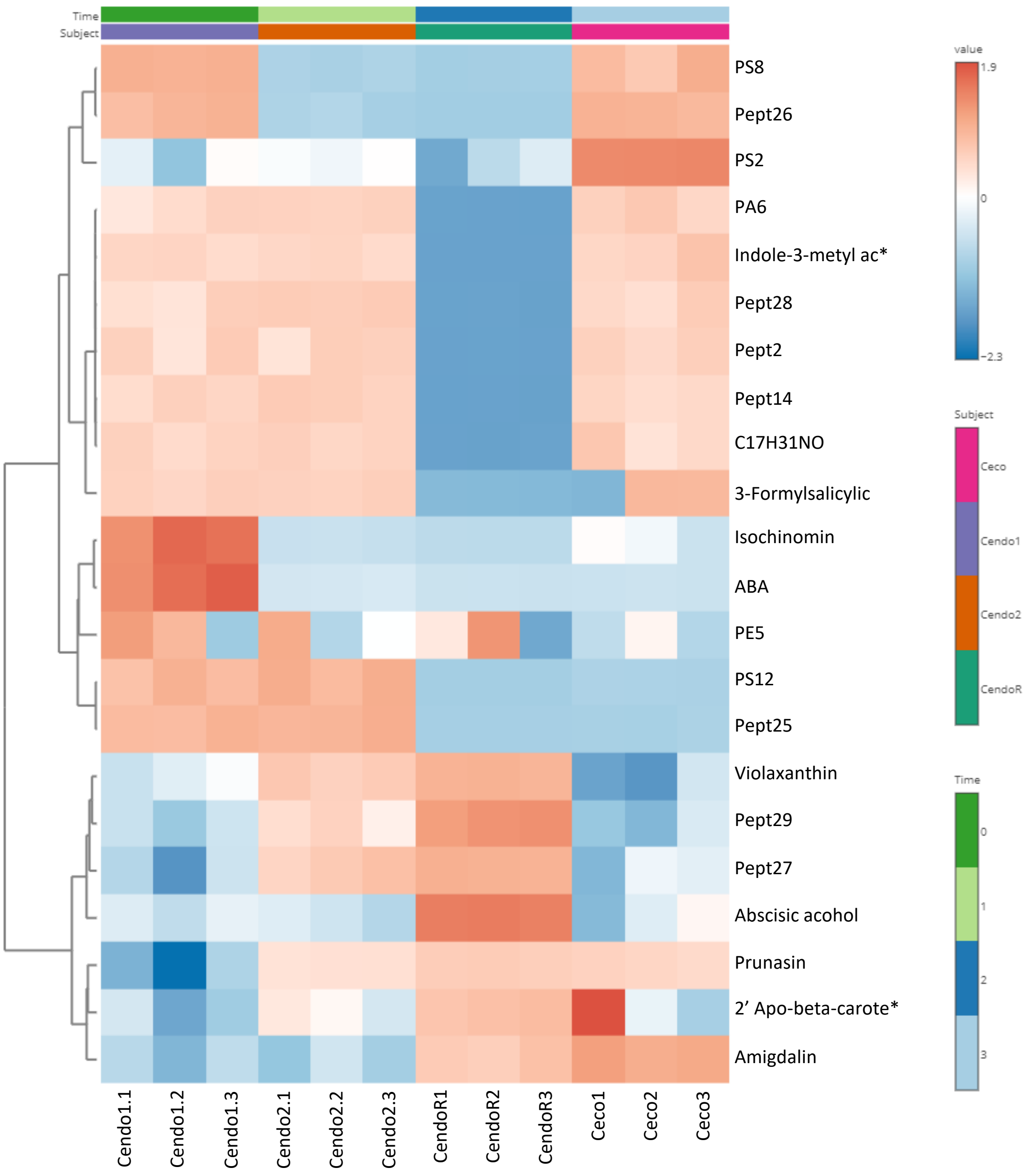

Supplementary figure S3. Heatmap of the metabolites that showed significant variations in control (C) trees during year 1. Cendo1: control untreated endodormant flower buds at the begging of the study, Cendo2: control endodormant flower buds at treatment date, CendoR: control endodormancy release flower buds and Ceco: control flower buds during ecodormancy. Three biological replicates were used for each group and are labeled as 1, 2 and 3. The metabolic group of each metabolite from the heatmap is detailed in Supplementary table S1.

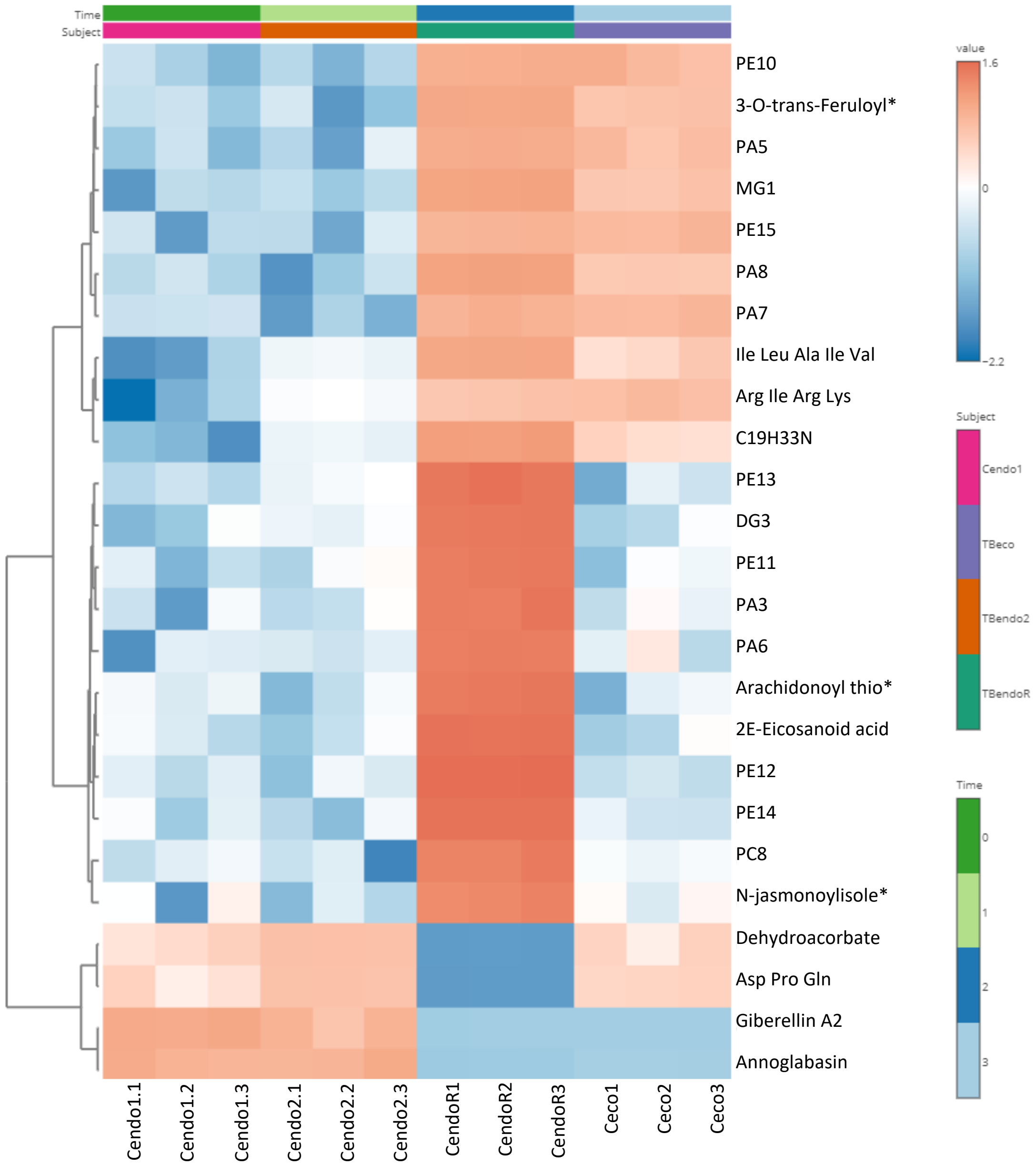

Supplementary figure S4. Heatmap of the metabolites that showed significant variations in trees treated with Broston® + Nitroactive® (TB) during year 1. Cendo1: control untreated endodormant flower buds at the begging of the study, TBendo2: treated endodormant flower buds at treatment date, TBendoR: treated endodormancy release flower buds and TBeco: treated flower buds during ecodormancy. Three biological replicates were used for each group and are labeled as 1, 2 and 3. The metabolic group of each metabolite from the heatmap is detailed in Supplementary table S1.

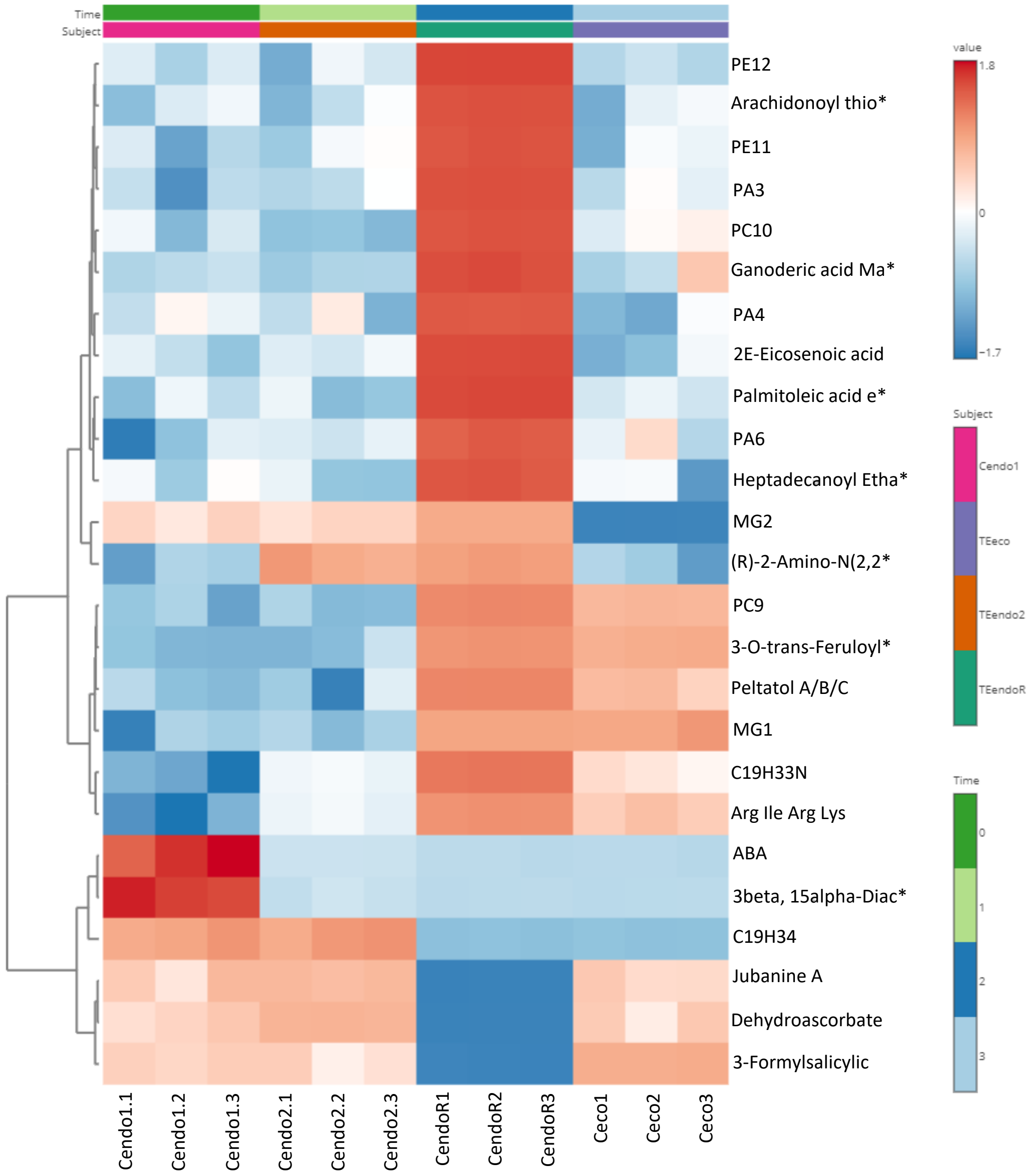

Supplementary figure S5. Heatmap of the metabolites that showed significant variations in trees treated with Erger® + Activ Erger® (TE) during year 1. Cendo1: control untreated endodormant flower buds at the begging of the study, TEendo2: treated endodormant flower buds at treatment date, TEendoR: treated endodormancy release flower buds and TEeco: treated flower buds during ecodormancy. Three biological replicates were used for each group and are labeled as 1, 2 and 3. The metabolic group of each metabolite from the heatmap is detailed in Supplementary table S1.

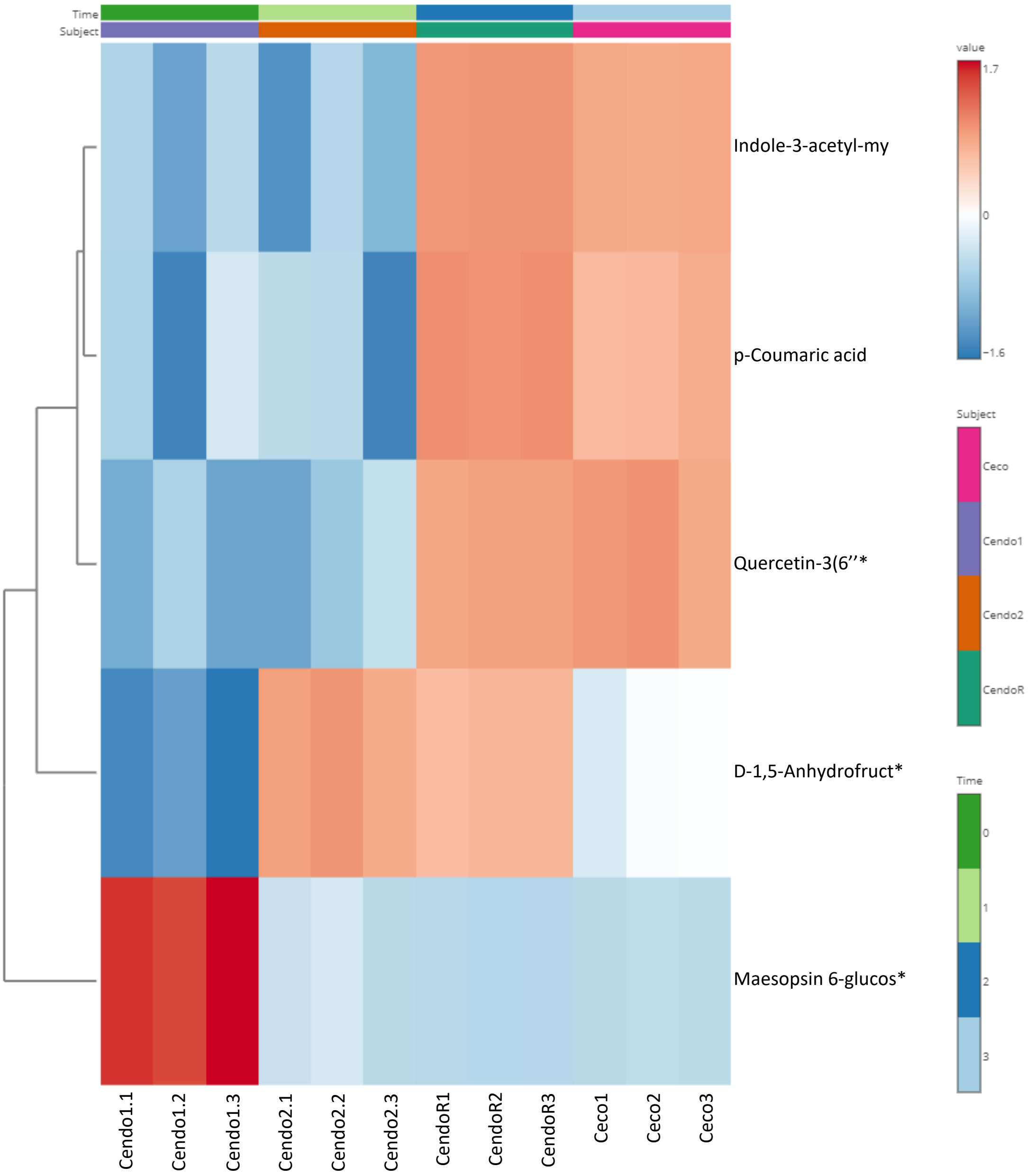

Supplementary figure S6. Heatmap of the metabolites that showed significant variations in control (C) trees during year 2. Cendo1: control untreated endodormant flower buds at the begging of the study, Cendo2: control endodormant flower buds at treatment date, CendoR: control endodormancy release flower buds and Ceco: control flower buds during ecodormancy. Three biological replicates were used for each group and are labeled as 1, 2 and 3. The metabolic group of each metabolite from the heatmap is detailed in Supplementary table S2.

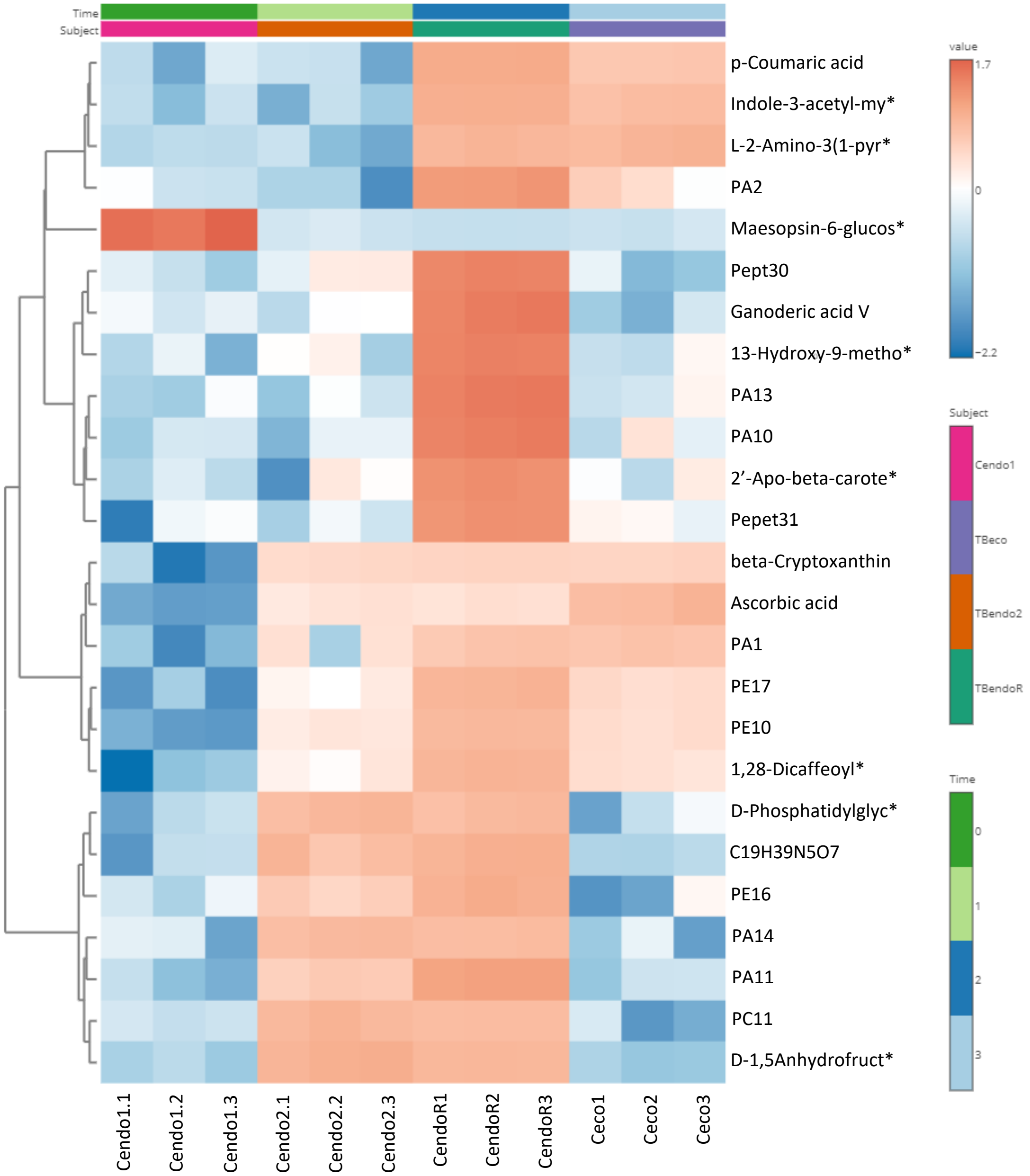

Supplementary figure S7. Heatmap of the metabolites that showed significant variations in trees treated with Broston® + Nitroactive® (TB) during year 2. Cendo1: control untreated endodormant flower buds at the begging of the study, TBendo2: treated endodormant flower buds at treatment date, TBendoR: treated endodormancy release flower buds and TBeco: treated flower buds during ecodormancy. Three biological replicates were used for each group and are labeled as 1, 2 and 3. The metabolic group of each metabolite from the heatmap is detailed in Supplementary table S2.

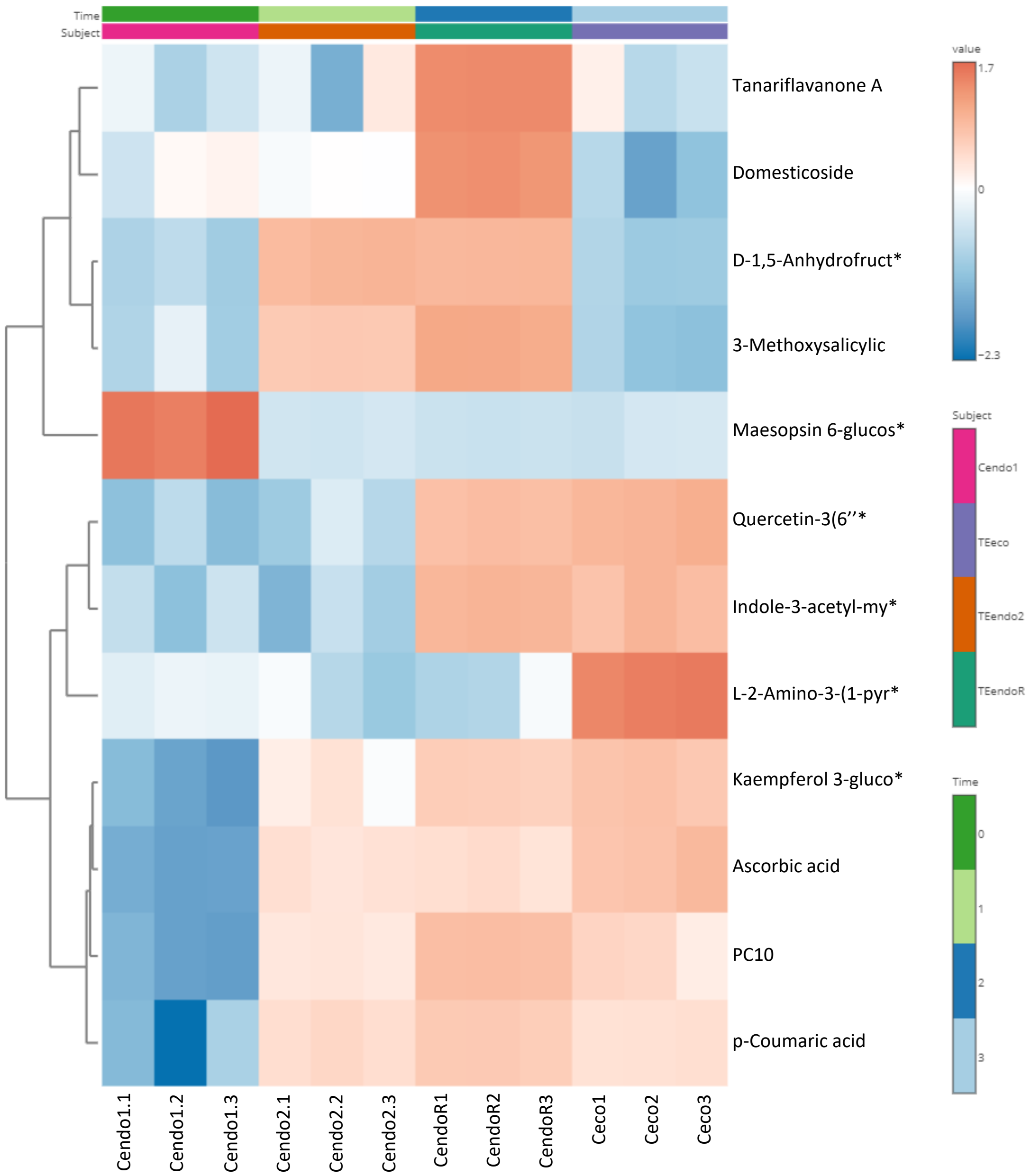

Supplementary figure S8. Heatmap of the metabolites that showed significant variations in trees treated with Erger® + Activ Erger® (TE) during year 2. Cendo1: control untreated endodormant flower buds at the begging of the study, TEendo2: treated endodormant flower buds at treatment date, TEendoR: treated endodormancy release flower buds and TEeco: treated flower buds during ecodormancy. Three biological replicates were used for each group and are labeled as 1, 2 and 3. The metabolic group of each metabolite from the heatmap is detailed in Supplementary table S2.
